# The mediating role of parent-child bonding for the prospective association of prenatal depressive symptoms with child development at 14 months postpartum

**DOI:** 10.1186/s12887-025-05730-5

**Published:** 2025-05-27

**Authors:** Ariane Göbel, Caroline Hilpert, Victoria Weise, Judith T. Mack, Susan Garthus-Niegel

**Affiliations:** 1 Institute for Systems Medicine (ISM), Faculty of Medicine, Medical School Hamburg MSH, Hamburg, Germany; 2 Department of Child and Adolescent Psychiatry and Psychotherapy, University Medical Center, Hamburg-Eppendorf, Hamburg, Germany; 3 Institute and Policlinic of Occupational and Social Medicine, Faculty of Medicine, Technische Universität Dresden, Dresden, Germany; 4 Department of Childhood and Families, Norwegian Institute of Public Health, Oslo, Norway

**Keywords:** DREAM study, Prenatal depression, Maternal mental health, Paternal mental health, Child development, Mother-child bonding, Father-child bonding, ASQ-3, EPDS, PBQ

## Abstract

**Background:**

Depressive symptoms in the perinatal period as well as difficulties developing an emotional bond towards the child have been described as potential risk factors for poor child development. Few studies have investigated the mediating role of parent-child bonding for the association between prenatal depressive symptoms and child outcomes. Research on this association is especially scarce regarding the paternal perspective. This study investigated the prospective association between both parents’ prenatal depressive symptoms and child development, taking the mediating role of parent-child bonding into account.

**Methods:**

Data of 1,178 mothers and 743 fathers were drawn from the prospective longitudinal cohort study “Dresden Study on Parenting, Work, and Mental Health” (DREAM). To investigate the prospective association between depressive symptoms during pregnancy and eight weeks postpartum (self-report, Edinburgh Postnatal Depression Scale), parent-child bonding at eight weeks postpartum (self-report, Postpartum Bonding Questionnaire), and child development at 14 months postpartum (parent-report, Ages and Stages Questionnaire-3), multiple regression and mediation analyses were conducted individually for both parents, including the confounders parental age, education, child’s sex assigned at birth, prematurity, and perceived social support.

**Results:**

In both parents, a statistically significant small-sized mediating effect of parent-child bonding for the association between prenatal depressive symptoms and child development was found, with higher depressive symptoms being associated with more parent-child bonding impairment, which was associated with poorer child development. Paternal depressive symptoms were not directly associated with child development, whereas higher levels of maternal prenatal depressive symptoms were directly associated with better child development at 14 months postpartum. After additionally controlling for postpartum depressive symptoms, the association between prenatal depressive symptoms and parent-child bonding was no longer significant, and a positive association between paternal prenatal depressive symptoms and child development emerged.

**Conclusions:**

Our results underline the importance of addressing depressive symptoms in the context of perinatal care to support parents experiencing mental health problems or struggles with the adjustment to parenthood early on. Future research on the complex dynamics of mental health, parent-child bonding, and child development is needed to replicate our findings. Our study highlights the relevance of including the perspective of both parents into research and clinical practice.

**Supplementary Information:**

The online version contains supplementary material available at 10.1186/s12887-025-05730-5.

## Background

Parental mental health problems can have a strong influence on family dynamics and relationships, as well as child development [1, 2]. A common mental health disorder is depression and thus also of high relevance in (expectant) parents [3]. Depressive symptoms with a significant impact on quality of life, can manifest in feelings of sadness or tearfulness, loss of energy and interest in everyday activities or hobbies, loss of energy, changes in sleep and appetite, concentration and memory problems and suicidal ideation [4]. In international studies, the overall prevalence rates for clinically relevant prenatal depression in mothers range from 15% for a major depression to 20.7% for any depression [5]. For fathers, international prevalence rates of 9.7% during pregnancy and 8.8% across the first year postpartum were identified [6]. These high prevalence rates of perinatal depression underline their individual and societal relevance, necessitating further research [7].

Previous literature clearly indicates a relationship between maternal depressive symptoms and negative child outcomes, like emotional and behavioral problems [8], poorer social-emotional, cognitive, language, motor, and adaptive behavior development [9, 10] with identified influences from infancy up into adulthood [11–13]. Maternal prenatal depressive symptoms have further been associated with neural development [14, 15]. Research on perinatal influences on child development has broadened its focus to include possibly relevant contributions from the second parents, in the majority of cases the father. In this context, influences through alterations in sperm epigenome (e.g., due to exposure to toxins, early life stress), and neuroendocrinological changes that might directly influence child development have been discussed, as well as indirect influences on their pregnant partner’s mental health or health behaviors, especially via own psychological functioning and mental health [16, 17]. While studies on paternal depressive symptoms during pregnancy indicate associations with poorer regulatory abilities in infancy [18], and more emotional and behavioral problems in later childhood [19], others report that prenatal depressive symptoms are of specific relevance for child negative affectivity in combination with subsequent parental postpartum depressive symptoms [20] and co-occurring perinatal paternal and maternal depressive symptoms [2]. Studies focusing on child cognitive development in the context of paternal depression are scarce and need further investigation [21]. For example, one study unexpectedly found that children of fathers reporting more prenatal depressive symptoms showed less behavior problems as well as a better cognitive performance when fathers reported co-occurring prenatal paternal anxiety [22]. These results highlight not only the importance of addressing mental health problems in both parents, but also the need to further investigate their longitudinal relevance for postpartum child developmental outcomes.

Depressive symptoms in parents might further influence maternal and paternal adjustment during the transition to parenthood in general, as well as the emotional bond towards the child [13, 23]. This parent-child bond, which starts to develop already during pregnancy, is an important aspect of the parent-child relationship and can be conceptualized as a subjective, unidirectional and parent-driven affective experience, manifesting in behavior and involving other cognitive (e.g. internal mental representation of the baby) and neurobiological aspects that are potentially important [24]. In severe cases of the parent struggling to develop an emotional bond towards their child, bonding disorders might be the consequence, manifesting in decreases in affection towards the child, rejection, pathological fear for the child, or anger [25]. Even though severe bonding disorders do not necessarily go along with perinatal depression [26], research indicates that mental health problems may negatively affect the developing parent-child relationship [27–32]. Again, here the maternal perspective has mainly been in the focus of research [33]. The few existing previous studies also highlight the importance of paternal postpartum [34–36] as well as prenatal depressive symptoms for father-child bonding [37, 38], while one study could not confirm such association between prenatal depressive symptoms and postpartum father-child bonding [39].

An impairment in parent-child bonding might negatively influence child development. Cross-sectional and longitudinal associations of mother-child bonding with child outcomes, which were mostly assessed via maternal report, and more rarely via observational measures or teacher report, have been found, like child temperament [40] as well as socio-emotional and behavioral development in infancy [40, 41], and early childhood [42, 43]. Paternal representations of the emotional relationship towards the child has further been associated with poorer father-child interaction and less developed vocabulary in the children at two years postpartum [44]. Given these results, investigating the mediating role of parent-child bonding eight weeks postpartum for the prospective association between prenatal depressive symptoms and child development 14 months postpartum can give further insight into the complex dynamics of the postpartum period. However, research including fathers is still clearly underrepresented within the field, even though involvement of fathers in childcare responsibilities has increased in recent years, along with changes in societal expectations regarding the paternal role [45, 46]. Including the paternal perspective is crucial to understand family dynamics and influencing factors for child development during the perinatal period. Further aspects might influence the associations described above. Next to individual (e.g., parental age, educational background), birth-related (e.g., preterm birth), or child-related characteristics (e.g., sex assigned at birth), personal relationships and the perception of support from these has been identified as factor associated with parental mental health in general, perinatal depression in particular [47–49], as well as parental adjustment, and more optimal parent-child bonding [50]. With social support being a protective factor against mental health problems in the peripartum period, it might therefore also hinder negative consequences of depressive symptoms on child development. Results from previous studies indicate that social support may be especially relevant for child development in high risk contexts [51]. Even independent of influencing parental mental health and adjustment, poor perceived partner or social support might negatively affect child development [52], as, for example, via influencing stress-related neuroendocrinological processes during pregnancy [53] or influences on parenting behavior [54].

In the first years of life, child development is characterized by constant changes, adaptive reactions, and the acquisition of new competences [55]. Rapid changes occur in physical, cognitive, language, and socioemotional development [56]. In addition to genetic predispositions, cognitive support, physical status, and nutrition, the social environment is a significant influencing factor for early child development [56, 57]. During early childhood, when the dependence on the caregivers is especially strong, stress within the family and poor relationship dynamics can have negative consequences for development [58]. Within the perspective of systemic family theory, wellbeing of one family member affects the wellbeing of the other family members as well as their interaction and relationship quality [59, 60]. This highlights the importance of understanding the experiences during the transition to parenthood and potential influences on child adjustment and developmental outcomes from the perspective of both caregivers.

## Methods

The aim of this study was to investigate the prospective association between parental prenatal depressive symptoms and child development at 14 months postpartum, taking into account a potential mediating role of parent-child bonding at eight weeks postpartum. For both parents, we expected a higher level of prenatal depressive symptoms to be directly associated with poor child development. Further, we expected a higher level of parental depressive symptoms to be associated with more parent-child bonding impairment, which in turn would to be negatively associated with child development. Finally, we tested for the stability of results when controlled for postpartum depressive symptoms.

### Participants

Data for this study were drawn from the Dresden Study on Parenting, Work, and Mental Health (“**DR**esdner **S**tudie zu **E**lternschaft, **A**rbeit und **M**entaler Gesundheit,” **DREAM**; [51, 61], an ongoing prospective longitudinal cohort study in (expectant) mothers and their partners in the area of Dresden, Germany (06/2017–ongoing). The DREAM study aim is to prospectively examine the association between parental work participation, role distribution, and stress across the perinatal period, including their effects on perinatal outcomes and family (mental) health. Recruitment started in 2017 and finished at the end of 2020. Currently the study consists of seven measurement points: T1 during pregnancy, T2 at 8 weeks after the anticipated birth date, T3 at 14 months, T4 at 2 years, T5 at 3 years, T6 at 4.5 years, and T7 at 7.5 years after birth. Participants comprise a community sample of *N* = 3,861 parents from Dresden, Germany and surroundings who are expecting a child and were mainly recruited at information events of obstetrical clinics. Pregnant women and their partners, residing in the Dresden area, with sufficient German skills to complete the study questionnaires were included. The DREAM study was approved by the Ethics Committee of the Technische Universität Dresden (No: EK 278062015). The parents were informed about the aims and procedures of the DREAM study and provided written informed consent. Detailed information on the design of the study can be found in the study protocol [61].

**Fig. 1:**
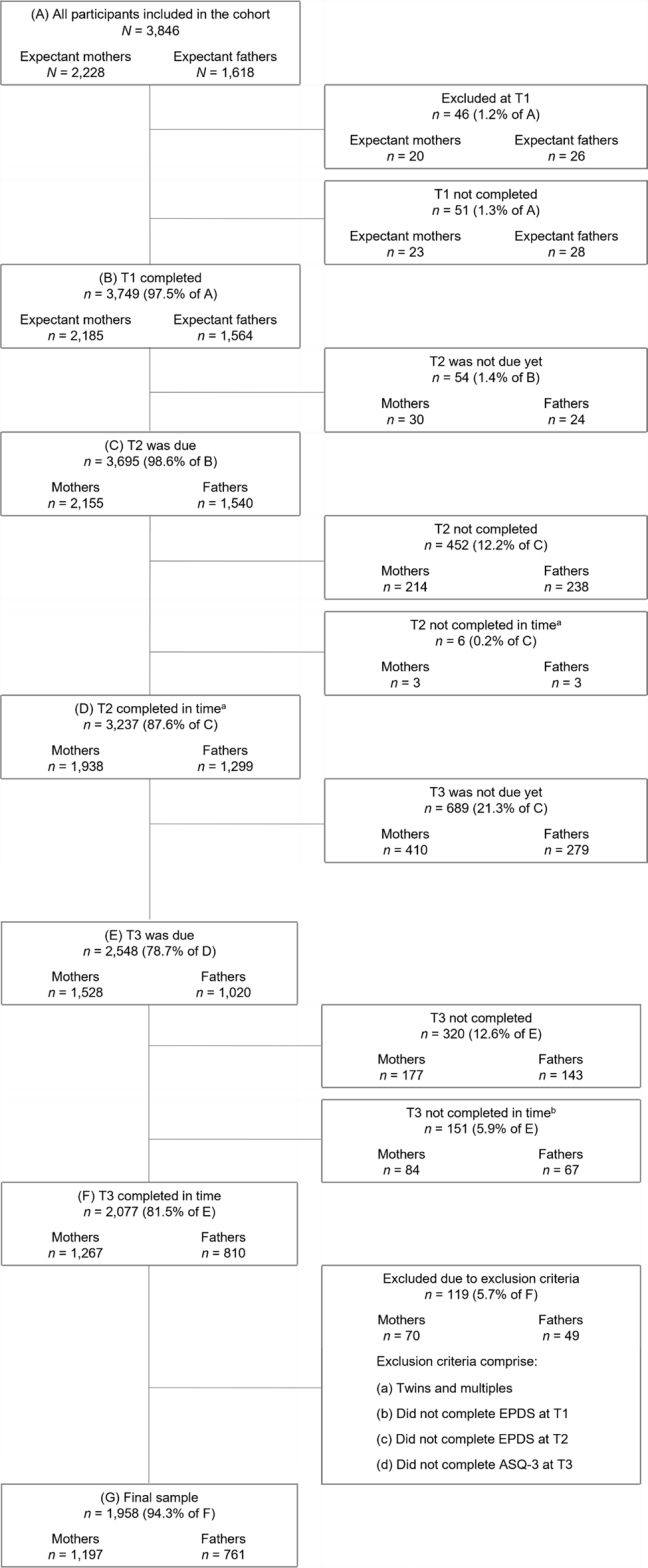
Flowchart of retention rate and exclusion criteria resulting in final sample. *Note*. Data from March 29th, 2021 (prospective data collection ongoing). T1: during pregnancy; T2: 8 weeks postpartum; T3: 14 months postpartum. EPDS: Edinburgh Postnatal Depression Scale; ASQ-3: Ages and Stages Questionnaire-3. ^a^Not within 12 and 16 months after the actual birth date. ^b^Not within 22 and 26 months after the actual birth date

The current study is based on version 8 of the quality-assured data files (date of extraction March 29th, 2021). For the aim of this study, data from (expectant) mothers and fathers from three assessment waves were included: during pregnancy (T1) as well as eight weeks after the anticipated birth date (T2) and 14 months postpartum (T3). Participants were included, if sufficient data from all three assessment points were available and if they filled out their questionnaire for T3 within 13 to 14 months postpartum. Further, for the context of this analysis, parents expecting more than one child were not included in this analysis (for details see Fig. 1).

### Measures

**Child development** was measured at T3 based on parental report with the Ages and Stages Questionnaire, Third Edition (ASQ-3; [62]). For the purpose of the DREAM study, the English version was translated into German and back-translated by a native speaker and adapted where necessary [61]. For this analysis, the ASQ-3 total score was used and calculated as a sum of the five subscales assessing age-appropriate development in the domains communication, personal-social, problem solving, fine and gross motor skills. Following the recommendations by Squires and Bricker [62], we recoded the items so that higher scores reflect a better child development, with a total score ranging from 0 to 300. For the purpose of this study, the total score was used, which showed good internal consistency for both mothers (α =.81) and fathers (α =.83).

**Depressive symptoms** at T1 and T2 were assessed with the German Version of the Edinburgh Postnatal Depression Scale (EPDS; [63, 64]). The ten items are answered on a 10-item 4-point Likert scale with a total score ranging from 0 to 30, and a higher score indicating higher levels of depressive symptoms. The internal consistency of the EPDS was good for both mothers (T1: α =.83, T2: α =.81) and fathers (T1: α =.81, T2: α =.80).

**Parent-child bonding** at T2 was assessed with the Postpartum Bonding Questionnaire (PBQ; [65, 66]), measuring the domains impaired bonding, rejection and anger, anxiety about care, as well as risk of abuse. The PBQ comprises 25 items, answered on a 6-point Likert scale, with a total score ranging from 0 to 125. Higher scores indicate more parent-child bonding impairment. The internal consistency of the PBQ was good for mothers (α =.89) and fathers (α =.84).

**Confounders** relevant for the current study comprised parental and child-related variables. The sociodemographic characteristics parental age and education (up to 10 years of school education = 0; more than 10 years of school education = 1) assessed at T1, and the child-related characteristics of child sex assigned at birth (female = 1; male = 2) and prematurity (0 = child being born before 37 completed weeks of gestation; 1 = child born at or after 37 completed weeks of gestation), each assessed at T2, were included. Further, perceived social support reported by both parents at T1 were assessed with the short form of the Perceived Social Support Questionnaire (Fragebogen zur sozialen Unterstützung, F-SozU K-14; [67, 68]), comprising 14 items answered on a 5-point Likert scale. For the total score, a mean value was calculated, with a possible range from 1 to 5 and a higher score indicating a higher level of perceived social support. The internal consistency was excellent for mothers (α =.92) and for fathers (α =.93).

### Statistical analysis

For the investigation of sample characteristics, descriptive statistics (means, standard deviations, percentages, where appropriate) were calculated. Attrition analyses based on independent *t*-tests and χ^2^ statistics were conducted to test for potential differences in sociodemographic characteristics and in the variables relevant for this study between parents who completed T3 vs. did not complete T3. Further, differences in the variables relevant for this study between parents participating together with their partner vs. those participating without a partner were investigated based on independent *t*-tests and χ^2^ statistics. Missing items on the psychometric scales up to 20% were substituted with the person’s mean score. For the ASQ-3, up to two missing items in each subscales can be replaced with mean values [62]. Outliers in ASQ-3 scores (deviating more than three standard deviations from the average) were excluded from further analysis (mothers: *n* = 30, fathers: *n* = 16). Bivariate associations among the included variables were calculated via Pearson correlations. To investigate the prospective association between prenatal depressive symptoms and child development at 14 months postpartum, separate multiple regression analyses were conducted for mothers and fathers. The confounders were dummy coded where necessary and added as independent variables using simultaneous forced inclusion. Effect sizes were interpreted using the standardized regression coefficients β according to the guidelines of Cohen (1988) [69], with a small effect being considered from β =.10, a moderate effect from β =.30, and a large effect from β =.50. Subsequently, parent-child bonding was added to the models as a mediator. Finally, to test whether associations of prenatal depressive symptoms with parent-child bonding and child development at 14 months postpartum would be significant above and beyond postpartum depressive symptoms, depressive symptoms at T2 were included. For mediation analysis, the PROCESS v3.5 macro in SPSS [60, 70] was used. Bootstrapping with 5,000 samples was applied to compute confidence intervals (95%) and inferential statistics. For all analyses, level of statistical significance was set to *p* <.05 (two-tailed).

## Results

### Sample characteristics

Sociodemographic and child-related characteristics as well as scores on the included measures of the final sample comprising of *N* = 1,178 mothers and *N* = 743 fathers are listed in Table 1. A high percentage were expecting their first child, which was in most cases born after week 37 of pregnancy. The sample was overall well educated, with 79% of mothers and 75% of fathers having an education level of more than ten years of school. Most participants were in a couple relationship. The paternal sample consisted mostly of participants who were included at T1 as the partner of a pregnant woman also enrolled in the study. In 27 cases (3.6%) of the final sample, fathers participated without their partner participating in the study. Also, 321 pregnant women (27.2%) were included at study intake without a partner also being enrolled. Thus, parent-reported data of 1,280 households was included in the study. Differences in the investigated parental variables between participants with and without a partner participating are listed in Supplement Tables 1 and 2. Mothers without partners participating in the study (NP) were significantly older than those with their partner participating (WP), with NP: *M* = 30.85 vs. WP: *M* = 30.00, *t*(529.46) = 3.58, *p* <.001. Mean levels of postpartum depression were significantly higher in the sample of mothers without participating partner than in mothers with participating partner (NP: *M* = 6.01 vs. WP: *M* = 5.50, *t*(537.89) = 0.21, *p* =.044), and perceived social support was significantly lower in the first group (NP: *M* = 4.27 vs. WP: *M* = 4.36, *t*(531.52) = -2.29, *p* =.024). No further significant differences were found.

**Table 1:** Descriptive statistics of the sample

	Mothers (*N* = 1,178)	Fathers (*N* = 743)
*Sample characteristics* ^*a*^		
Parental age in years at T1, *M* ± *SD* (min-max)	30.2 ± 3.9 (15–43)	32.4 ± 4.8 (20–56)
Week of pregnancy at T1, *M* ± *SD* (min-max)	29.9 ± 6.2 (8–41)	
Age of the child at T2 in weeks, *M* ± *SD* (min-max)	8.5 ± 2.2 (3–24)	8.9 ± 2.2 (4–21)
Age of the child at T2 in months, *M* ± *SD* (min-max)	13.7 ± 0.5 (13–14)	13.8 ± 0.4 13–14)
Citizenship, *n*^*b*^ (%)^c^		
German	1,145 (97.2)	735 (98.9)
Other country	33 (2.8)	8 (1.1)
Country of birth, *n*^*b*^ (%)^c^		
Germany	1,129 (96.2)	725 (98.1)
Other country	45 (3.8)	14 (1.9)
Native language, *n*^*b*^ (%)^c^		
German	1,121 (96.3)	718 (98.2)
Other language	31 (2.7)	7 (1.0)
Both German and another language	12 (1.0)	6 (0.8)
Education, *n*^*b*^ (%)^c^		
≤ 10 years of schooling	240 (20.4)	184 (25.0)
> 10 years of schooling	938 (79.6)	552 (75.0)
Relationship status, *n*^*b*^ (%)^c^		
In a relationship	1,160 (99.0)	736 (99.9)
Not in a relationship	12 (1.0)	1 (0.1)
Number of previous children, *n*^*b*^ (%)^c^		
0	931 (79.8)	579 (80.0)
1	200 (17.1)	114 (15.8)
2	29 (2.5)	23 (3.2)
3	6 (0.5)	5 (0.7)
4	1 (0.1)	2 (0.3)
Child sex assigned at birth^d^, *n*^*b*^ (%)^c^		
Female	604 (51.9)	370 (51.2)
Male	559 (48.1)	353 (48.8)
Prematurity, *n*^*b*^ (%)^c^		
Yes	52 (4.4)	30 (4.0)
No	1,126 (95.6)	713 (96.0)
*Psychometric questionnaires*		
Prenatal depressive symptoms (EPDS, T1; 0–30), *M* ± *SD* (min-max)	5.4 ± 3.9 (0–23)	3.7 ± 3.5 (0–23)
Postpartum depressive symptoms (EPDS, T2; 0–30), *M* ± *SD* (min-max)	5.6 ± 3.8 (0–25)	3.4 ± 3.2 (0–25)
Parent-child bonding (PBQ, T2; 0–125), *M* ± *SD* (min-max)	12.8 ± 9.8 (0–93)	12.4 ± 8.0 (0–50)
Child development (ASQ-3, T3; 0–300), *M* ± *SD* (min-max)	229.0 ± 40.1 (95–300)	224.8 ± 42.2 (95–300)
Perceived social support (F-SozU K-14, T1; 0–5), *M* ± *SD* (min-max)	4.3 ± 0.6 (1.1–5)	4.2 ± 0.6 (1.7–5)

*Note*. T1: during pregnancy; T2: 8 weeks postpartum; T3: 14 months postpartum; EPDS: Edinburgh Postnatal Depression Scale; PBQ: Postpartum Bonding Questionnaire; ASQ-3: Ages and Stages Questionnaire-3; F-SozU K-14: 14-item short form of the Perceived Social Support Questionnaire (Fragebogen zur sozialen Unterstützung). ^a^If not stated otherwise, information assessed at T1. ^*b*^Slightly varies due to missing data of some participants. ^c^Valid percentages reported. ^d^Assessed at T2

**Table 2:** Bivariate associations among the included variables

		1	2	3	4	5	6	7	8	9
Mothers	1. Prenatal depressive symptoms (EPDS, T1)	1								
	2. Postpartum depressive symptoms (EPDS, T2)	.438***	1							
	3. Parent-child bonding (PBQ, T2)^a^	.158***	.429**	1						
	4. Child development (ASQ-3, T3)	.037	−.034	−.097***	1					
	5. Parental age	−.035	−.048	−.024	−.129***	1				
	6. Education	−.117***	−.028	.134***	.016	.060*	1			
	7. Child sex assigned at birth	−.035	−.013	−.010	−.080**	.058	−.026	1		
	8. Prematurity	.059*	.058*	.019	−.153***	.059*	−.035	.025	1	
	9. Perceived social support (F-SozU K-14, T2)	−.306**	−.278***	−.187***	.083**	−.014	.080**	.037	.018	1
Fathers	1. Prenatal depressive symptoms (EPDS, T1)	1								
	2. Postpartum depressive symptoms (EPDS, T2)	.540***	1							
	3. Parent-child bonding (PBQ, T2) ^a^	.227***	.389***	1						
	4. Child development (ASQ-3, T3)	−.019	−.115**	−.137***	1					
	5. Parental age	.006	−.082*	−.077*	−.038	1				
	6. Education	−.044	−.026	.108**	.033	−.040	1			
	7. Child sex assigned at birth (T2)	.006	−.013	−.013	−.131***	.028	−.019	1	1	
	8. Prematurity	−.002	.031	−.011	−.159***	.041	−.012	−.002	1	
	9. Perceived social support (F-SozU K-14)	−.309***	−.220***	−.182***	.123***	−.131***	.036	.070	.006	1

*Note*. EPDS: Edinburgh Postnatal Depression Scale; PBQ: Postpartum Bonding Questionnaire; ASQ-3: Ages and Stages Questionnaire-3; F-SozU K-14: 14-item short form of the Perceived Social Support Questionnaire (Fragebogen zur sozialen Unterstützung); T1: during pregnancy; T2: 8 weeks postpartum; T3: 14 months postpartum. ^a^A higher score in parent-child bonding indicates more bonding impairment. * *p* <.05; ** *p* <.01; *** *p* <.01

### Attrition analysis

Next, sample characteristics were compared between the parents who completed T3 (C) vs. did not complete T3 (NC). Parents were more likely to report a higher level of education of more than ten years of school education if they completed questionnaires at all three assessment time points compared to non-completers, both in mothers (C: 79.5% vs. NC: 67.4%; *χ*^*2*^(1, *n* = 1683) = 20.31, *p* <.001) and in fathers (C: 71.8% vs. NC: 62.7%; *χ*^*2*^(1, *n* = 1203) = 8.58, *p* =.003). Mean values for prenatal depressive symptoms were significantly lower for completing mothers (C: *M* = 5.50 vs. NC: *M* = 6.36; *t*(401) = -2.97, *p* =.003) and fathers (C: *M* = 3.82 vs. NC: *M* = 4.30; *t*(1204) = -1.97, *p* =.049) than for non-completers. No further significant differences were identified (see Supplement Tables 3 and 4 for details).

**Table 3:** Multiple regression analysis of the included variables on child development

	Mothers						Fathers					
	B	SE B	β	95% CI		*p*	B	SE B	β	95% CI		*p*
				lower	upper					lower	upper	
Prenatal depressive symptoms (EPDS, T1)	0.90	0.31	.09	0.29	1.50	.004	0.92	0.48	.08	-0.02	1.85	.054
Parent-child bonding^a^ (PBQ, T2)	-0.41	0.12	−.10	-0.65	-0.17	<.001	-0.65	0.20	−.12	-1.04	-0.26	<.001
Parental age	-1.18	0.30	−.12	-1.76	-0.60	<.001	-0.32	0.32	−.04	-0.95	0.32	.325
Education	4.09	2.94	.04	-1.67	9.85	.164	0.99	3.60	.01	-6.07	8.05	.783
Child sex assigned at birth	-5.22	2.30	−.07	-9.74	-0.70	.024	-12.13	3.07	−.15	-18.15	-6.12	<.001
Prematurity	-25.28	5.68	−.13	-36.42	-14.15	<.001	-35.27	7.81	−.17	-50.60	-19.94	<.001
Perceived social support (F-SozU K-14, T3)	5.82	2.15	.08	1.60	10.04	.007	8.42	2.63	.13	3.26	13.57	<.001

*Note*. EPDS: Edinburgh Postnatal Depression Scale; PBQ: Postpartum Bonding Questionnaire; ASQ-3: Ages and Stages Questionnaire-3; F-SozU K-14: 14-item short form of the Perceived Social Support Questionnaire (Fragebogen zur sozialen Unterstützung); T1: during pregnancy; T2: 8 weeks postpartum; T3: 14 months postpartum. ^a^A higher score in parent-child bonding indicates more bonding impairment

**Table 4:** Multiple regression analysis of the included variables on child development, including depressive symptoms at T2

	Mothers						Fathers					
	B	SE B	β	95% CI		*p*	B	SE B	β	95% CI		*p*
				lower	upper					lower	upper	
Prenatal depressive symptoms (EPDS, T1)	0.83	0.35	.08	0.14	1.52	.018	1.56	0.58	.12	0.42	2.69	.007
Parent-child bonding^a^ (PBQ, T2)	-0.30	0.14	−.07	-0.57	-0.02	.036	-0.60	0.22	−.11	-1.04	-0.16	.008
Parental age	-1.35	0.32	−.12	-1.97	-0.73	<.001	-0.42	0.35	−.05	-1.10	0.25	.221
Education	6.32	3.11	.06	0.22	12.42	.042	1.51	3.84	.01	-6.02	9.04	.694
Child sex assigned at birth	-5.89	2.45	−.07	-10.69	-1.09	.016	-13.17	3.27	−.15	-19.59	-6.75	<.001
Prematurity	-34.87	5.77	−.17	− 46.20	-23.55	<.001	-34.06	8.29	−.15	-50.32	-17.79	<.001
Perceived social support (F-SozU K-14, T2)	5.17	2.30	.07	0.66	9.67	.025	9.17	2.78	.13	3.72	14.62	.001
Postpartum depressive symptoms (EPDS, T2)	-0.10	0.39	−.01	-0.88	0.67	.791	-1.38	0.64	−.10	-2.64	-0.12	.033

*Note*. EPDS: Edinburgh Postnatal Depression Scale; PBQ: Postpartum Bonding Questionnaire; ASQ-3: Ages and Stages Questionnaire-3;

F-SozU K-14: 14-item short form of the Perceived Social Support Questionnaire (Fragebogen zur sozialen Unterstützung). T1: during pregnancy; T2: 8 weeks postpartum; T3: 14 months postpartum. ^a^A higher score in parent-child bonding indicates more bonding impairment

### Bivariate associations among study variables

Bivariate associations among the included variables are listed in Table 2. There was no significant bivariate prospective association between maternal prenatal depressive symptoms and mother-reported child development. However, there was a significant, small-sized positive prospective association of higher levels of maternal prenatal depressive symptoms with more mother-child bonding impairment. Further, there was a significant small-sized negative prospective association of more mother-child bonding impairment and poorer child development.

For the paternal sample, there was also no significant prospective association between prenatal depressive symptoms and child development. However, as in the maternal sample, prenatal depressive symptoms had a significant small-sized, positive prospective association with father-child bonding. Thus, fathers reporting higher levels of depressive symptoms also reported more father-child bonding impairment. Finally, a small-sized, negative prospective association between father-child bonding and child development was found.Fathers reporting more bonding impairment also reported their child to have a poorer developmental outcome.

We further explored the correlations in investigated variables, including confounders, among participating couples. In the participating couples, correlations between partners were small-to medium-sized for concurrent depression scores as well as for parent-child bonding. Correlations between the maternal and paternal evaluation of child development were large-sized (for details, see Supplement Table 5).

### Multiple regression analysis

Next, multiple regression analyses including confounders were conducted separately for mothers and fathers (Table 3). The included variables significantly predicted child development in both regression analysis for mothers, with *R*² =.07, *F*(7,1152) = 12.11, *p* <.001, and fathers, with *R*² =.08, *F*(7,710) = 8.74, *p* <.001. A higher level of maternal prenatal depressive symptoms was prospectively associated with more optimal child development at 14 months postpartum (β =.09, *p* =.004). For fathers, there was no significant prospective association between prenatal depressive symptoms and child development at 14 months postpartum (β =.08, *p* =.054). The negative prospective association between mother-child bonding and child development was still significant after controlling for confounders, with more parent-child bonding impairment being associated with poorer child development, for both mothers (β = −.10, *p* <.001) and fathers (β = −.12, *p* <.001). Among the confounding variables, prematurity and child sex assigned at birth were negatively associated with child development evaluated by both mothers and fathers. Those being born premature showed poorer child development in the maternal (β = −.13, *p* <.001) and paternal analysis (β = −.17, *p* <.001). Also, children assigned male sex at birth showed poorer child development evaluated by mothers (β = −.07, *p* =.024) and fathers (β = −.15, *p* <.001). Higher perceived social support in mothers (β =.08, *p* =.007) and fathers (β =.13, *p* <.001) was significantly associated with more optimal child development.

To test whether the reported associations would be significant above and beyond postpartum depressive symptoms, postpartum depressive symptoms at T2 were included in the regression analyses in an additional step (Table 4). Including postpartum depressive symptoms did not significantly change the amount of explained variance for child development in the maternal sample, with *R*² ∆=.00, *F*(1,1151) = 0.07, *p* =.791. For the paternal sample, including postpartum depressive symptoms significantly increased the amount of explained variance in child development, with *R*² ∆ =.01, *F*(1,709) = 0.03, *p* =.033. The previously reported prospective associations stayed significant after inclusion of postpartum depressive symptoms. For the paternal sample, the prospective association between prenatal depressive symptoms and child development at 14 months postpartum turned significant (β =.12, *p* =.007).

### The mediating role of parent-child bonding

**Fig. 2:**
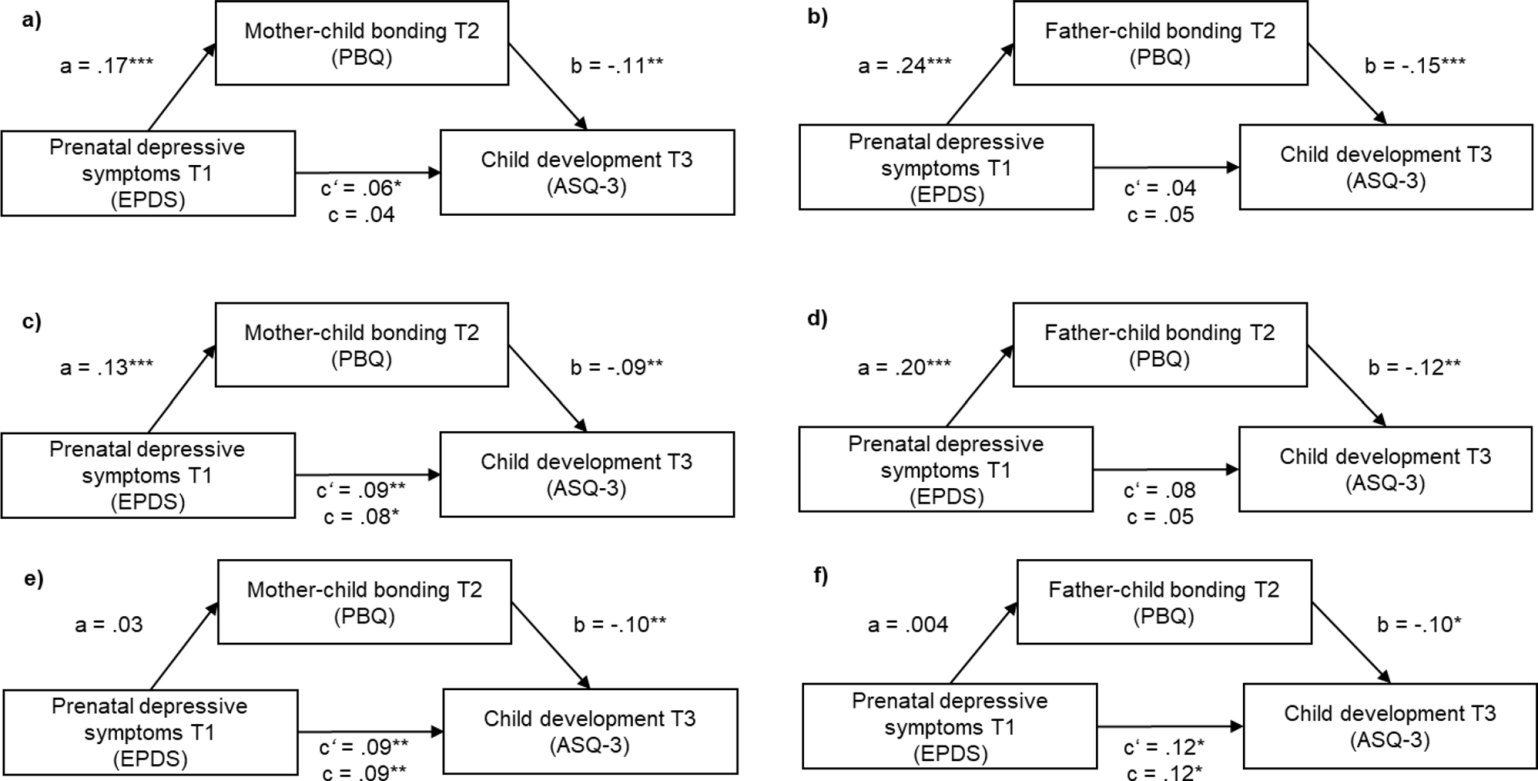
Mediation analysis of prenatal depressive symptoms via parent-child bonding eight weeks postpartum on child development 14 months postpartum. *Note*. Controlled for the confounders parental age, education, child sex assigned at birth, and prematurity (**a**, **b**), additionally controlled for perceived social support (**c**, **d**) and tested for stability of effects after inclusion of postpartum depressive symptoms (**e**, **f**). EPDS: Edinburgh Postnatal Depression Scale; PBQ: Postpartum Bonding Questionnaire; ASQ-3: Ages and Stages Questionnaire-3; T1: during pregnancy; T2: 8 weeks postpartum; T3: 14 months postpartum. * *p* <.05. ** *p* <.01. *** *p* <.001

In order to examine whether parent-child bonding mediated the prospective association between parental prenatal depressive symptoms and child development, mediation analyses were conducted. First, mediation analyses were conducted including the confounding variables except perceived social support (Fig. 2). For mothers, there was a significant positive prospective association between depressive symptoms and the mediator mother-child bonding (a =.17, *p* <.001), which in turn was negatively associated with child development (b = −.11, *p* =.001). The indirect path between depressive symptoms and child development was significant, with a small-sized, negative effect (ab = −.02, 95% BCa CI [-0.03; -0.08]). Thus, a higher level of prenatal depressive symptoms was prospectively associated with more mother-child bonding impairment, which in turn was prospectively associated with poorer child development. The direct effect of prenatal depressive symptoms on child development was still significant (c’ =.06, *p* <.05) and therefore only partially mediated by mother-child bonding.

Next, mediation analysis after controlling for all confounding variables including perceived social support were conducted (Fig. 2). There was a significant positive prospective association between depressive symptoms and the mediator mother-child bonding (a =.13, *p* <.001) as well as a significant negative association between mother-child bonding and child development (b = −.10, *p* <.05). The indirect path between depressive symptoms and child development was significant, with a small-sized, negative effect (ab = −.02, 95% CI [-0.03; -0.01]). Thus, a higher level of prenatal depressive symptoms was prospectively associated with more mother-child bonding impairment, which in turn was prospectively associated with poorer child development. The direct effect of prenatal depressive symptoms on child development was still significant (c’ =.09, *p* <.05) and therefore only partially mediated by mother-child bonding. Finally, to test whether the effects would still be significant after including postpartum depressive symptoms, mediation analysis with all confounding variables was repeated with additionally including postpartum depressive symptoms at T2. The prospective association between depressive symptoms and the mediator mother-child bonding was no longer significant (a = −.03, *p* =.284) as was the indirect path between prenatal depressive symptoms and child development (ab =.03, 95% BCa CI [-0.03; 0.12]). The prospective negative association between mother-child bonding and child development (b = −.10, *p* <.01) and the direct effect of prenatal depressive symptoms on child development (c’ =.09, *p* <.01) remained significant. Thus, when controlling for postpartum depressive symptoms, the prospective association of prenatal depressive symptoms with child development was no longer mediated by mother-child bonding. However, the individual prospective associations of prenatal depressive symptoms and mother-child bonding with child development remained significant.

For fathers, after including the confounding variables except for perceived social support, prenatal depressive symptoms were positively associated with father-child bonding (a =.24, *p* <.001), which in turn were negatively associated with child development (b = −.15, *p* <.001). This resulted in a significant indirect effect between depressive symptoms and child development (ab = −.04, 95% BCa CI [-0.06; -0.02]). The total effect of prenatal depressive symptoms with child development was not significant (c =.05, *p* =.906). Fathers reporting higher levels of prenatal depressive symptoms also reported more father-child bonding impairment, which in turn predicted poorer child development. After controlling for all confounding variables including perceived social support, the positive association between prenatal depressive symptoms and father-child bonding (a =.20, *p* <.001), as well as the negative association with child development (b = −.12, *p* <.001) remained significant, with a significant indirect effect between depressive symptoms and child development (ab = −.03, 95% BCa CI [-0.04; -0.01]). The total effect (c) of prenatal depressive symptoms with child development was again not significant. Fathers reporting higher levels of prenatal depressive symptoms also reported more father-child bonding impairment, which in turn predicted poorer child development. In the subsequent analysis including postpartum depressive symptoms, prenatal depressive symptoms were not associated with father-child bonding (a =.004, *p* =.926). However, father-child bonding was still negatively associated with child development (b = −.10, *p* <.05). The indirect effect between depressive symptoms and child development was not significant (ab =.004, 95% BCa CI [-0.10; 0.01]). In this model, the total effect of prenatal depressive symptoms with child development was significant (c =.12, *p* <.05). Fathers reporting more father-child bonding impairment reported poorer child development at 14 months postpartum. As the direct effect c’ was also significant, fathers with higher levels of prenatal depressive symptoms gave higher scores in child development.

### Explorative analysis

To further investigate the nature of effect of perceived social support in the mediation model, we explored its potential moderation for the significant paths in the mediation model after including depressive symptoms, in particular for the prospective association of parent-child bonding (b-path) and prenatal depressive symptoms (c’-path) on child development. As in the maternal model controlling for postpartum depressive symptoms, the indirect effect was no longer significant, consequently in this analysis, the index for this moderated mediation was also not significant (*B* = 0.00, *SE B* = 0.02, 95% BCa CI [-0.43; 0.37]). Further, no significant interaction was found between either mother-child bonding and perceived social support (*B* = 0 0.09, *SE B* = 0.20; 95% BCa CI [ -0.31; 0.50]) or prenatal depressive symptoms and perceived social support (*B* = 0.13, *SE B* = 0.44, 95% BCa CI [− 0.74; 0.99]). For fathers, as there was also no significant indirect mediation after controlling for depressive symptoms postpartum, there was no significant moderated mediation (*B* = -0.02, *SE B* = 0.05, 95% BCa CI [-0.15; 0.50]). The interaction terms indicate that there was no significant moderation for the prospective association of father-child bonding (*B* = -0.22, *SE B* = 0.29, 95% BCa CI [-0.795; 0.35]) or prenatal depressive symptoms (*B* = -0.82, *SE B* = 0.68, 95% BCa CI [ -2.15; 0.52]) with child development.

## Discussion

The aim of the current study was to investigate the prospective association between maternal and paternal prenatal depressive symptoms with parent-reported child development at 14 months postpartum, taking into account the mediating role of parent-child boding at eight weeks postpartum.

In line with our hypothesis, for both the maternal and paternal sample after controlling for parental age and education, child sex assigned at birth, prematurity, and perceived social support, a higher level of prenatal depressive symptoms was significantly prospectively associated with more parent-child bonding impairment at eight weeks postpartum, which in turn was significantly associated with poorer parent-reported child development at 14 months postpartum. In both analyses, the strength of the effects was small-sized. The associations are in line with previous literature indicating that parents experiencing depressive symptoms might struggle with developing an emotional bond to their child [25, 28–30]. Depressive symptoms like increased feelings of sadness, reduced joy or interest might interfere with experiencing and developing an emotional relationship towards the child. However, after controlling for postpartum depressive symptoms, the prospective association between prenatal depressive symptoms and parent-child bonding was no longer significant, which might be explained by the stronger association between parent-child bonding with concurrent depressive symptoms, which was also evident in the bivariate analysis among variables. However, our results indicate that prenatal depressive symptoms show prospective relevance for the experience of parenthood in the postpartum period [37, 38]. Previous depressive symptoms are an important predictor of postpartum depression [71], which is also indicated by the correlation found in our study between depressive symptoms assessed during pregnancy and at eight weeks postpartum. Thus, identifying these symptoms already during pregnancy is important to offer best support and initiate preventive measures at an early stage. As only a few studies have been published so far on prenatal mental health influence on postpartum adjustment in fathers, our analyses address current gaps in the literature and underline the importance of focusing on both parents’ mental health already during pregnancy.

Further, the significant negative associations between parent-child bonding impairment at eight weeks postpartum and child development twelve months later add to the literature on the influence of parent-child bonding on child outcomes [40–43]. Parent-child bonding can be understood as the subjective, emotional aspect of the developing parent-child relationship and thus as related to parent-child interaction [24]. Therefore, one explanation for the observed associations might be that parents reporting poorer emotional parent-child bonding might engage with their child less often in a way that supports child development. As previous literature highlighted that not only maternal but also paternal involvement in childcare supports child developmental outcomes [72, 73], our results underline the relevance of better understanding the developing father-child bond, potential specific qualities of this concept in fathers [33], and the influence on child-development.

Regarding the prospective association of prenatal depressive symptoms with child development, results differed for mothers and fathers and were not consistent with our hypothesis. For fathers, no significant association was found in our main analysis, whereas for mothers an unexpected positive prospective association was observed, indicating that those reporting higher levels of prenatal depressive symptoms also reported better child development at 14 months postpartum. After controlling for postpartum depressive symptoms, the direct effect of prenatal depressive symptoms on child development turned significant for fathers as well, indicating a suppression effect. A suppression effect occurs when inclusion of a third variable increases or even changes the direction of effects between two variables. Thus, after controlling for the relevance of postpartum depressive symptoms, the individual contribution of prenatal depressive symptoms emerged. These results indicate that depressive symptoms at different stages during the peripartum period might have divergent effects on parental adjustment and child development. Literature has highlighted the relevance of maternal prenatal depressive symptoms as a risk factor for child development regarding neuroendocrinological processes, health-related behavior, birth outcomes, but also for the adjustment during the transition to motherhood (e.g., perceived self-efficacy in childcare, maternal-fetal bonding) [28, 74], which might in turn, and potential in interplay with depressive symptoms postpartum, influence adjustment after childbirth, parenting behavior, and the quality of the mother-child interaction, which have all individually been identified as influencing factors for child development [7, 23, 42, 44, 75]. Paternal mental health independently influences child development. Potential influences could already be relevant prenatally, as health related behavior or external stressors and toxins might influence the sperm epigenome [76]. Further, in comparison to fathers without depressive symptoms, those with depressive symptoms might struggle to adapt to pregnancy and to offer support to their partner [23]. Even though the association between both parents’ concurrent level of depressive symptoms was also small-sized in our study, it still indicates that a negative emotional state of one parent is associated with the other one’s. In the postpartum period, depressive symptoms could influence parenting behavior, self-efficacy in parenting, and the developing relationship with the child also in fathers [60]. The positive association between parental prenatal depressive symptoms and better parent-reported child development is not in line with our hypothesis. Jones et al. (2023) [22] also unexpectedly found that paternal depressive symptoms were associated with fewer behavioral problems and higher IQ scores at age 6–8. They argue that the parenting of one parent, for example the father, might buffer against the effects of the other one’s mental health problems on child development. Intensity of depressive symptoms might be relevant in this context, as in our community sample the average depressive symptom levels were rather low in both mothers and fathers compared to clinically relevant depression levels, which might not affect functionality in such a severe way that it negatively influences parenting or the parent-child interaction. As heterogeneous trajectories of depressive symptom levels across the perinatal period have been identified [77], some parents might also experience a decline in depressive symptoms postpartum. Additionally, some parents with higher levels of perinatal depressive symptoms might have dropped out of the study, as the attrition analysis indicated. This might further influence the observed associations due to a reduced variance in the analyzed data.

The transition to parenthood is a complex interplay between both risk and protective factors. One potential protective factor is perceived social support from friends and family, which could form a buffer between mental health problems and child developmental outcomes [65]. For example, in families with more perceived social support, children might have more interaction with other persons taking care of them, which might have a positive influence on child development. In line with this, in our regression analysis the confounder perceived social support was positively associated with more optimal child development. Also, a comparison between mediation models before and after controlling for perceived social support showed that for mothers, the direct effect of prenatal depressive symptoms turned significant only after including perceived social support, indicating a suppressor effect of social support on the association between prenatal depressive symptoms and child development. However, as shown in our explorative analysis, the prospective association of mother-child bonding and prenatal depressive symptoms with child development was not contingent on the level of perceived social support. Longitudinal analyses are needed to investigate the unexpected positive association of parental depressive symptoms with child development, taking additional potentially relevant variables into account to disentangle dynamics in family mental health during the transition to parenthood.

### Strength and limitations

The strengths of this study are the large sample size and prospective study design, allowing the investigation of relations among the variables across the perinatal period as well as taking into account potential confounding variables. Also, this is one of the few studies investigating the mediating role of parent-child bonding for child development in both mothers and fathers. However, several limitations need to be considered. In terms of generalizability, it should be noted that the participants were overall well educated and in a relationship. The significant results in our mediation models however highlight that the observed associations are relevant also in presumably low-risk samples. Most participants were born and raised in Germany, which limits generalizability of results. As most studies have focused on socio-emotional development as outcome for child development, our study adds important insights into child development comprising gross and fine motor, language, problem-solving, and socio-emotional skills. Here, we included each parent’s individual evaluation of child development in comparison to many studies relying on maternal report only. Even though parent-report is regularly used in assessing child development in diverse domains and can give insight into everyday life beyond performance of a child in the laboratory, it is never possible to rule out a potential reporting bias compared to observational measures. It is a limitation of this study that the German version of the ASQ-3 we used has not been validated yet. Also, unlike the original version of the ASQ-3, our instructions did not include a directive for parents to actively assess their child’s ability to perform the tasks outlined in the questionnaire items. This modification was implemented for the purpose of our study, to enable parents to fill out our questionnaires without the child necessarily being around. Even though maternal and paternal ratings were provided independently, the strong correlation between their evaluations suggests a high degree of consistency. However, we acknowledge that this departure from the standard protocol may have led to a more subjective perception of the child’s competencies provided by parents, thereby warranting caution in the interpretation of the findings.

## Conclusion

This study highlights the mediating role of parent-child bonding for the prospective association between prenatal depressive symptoms and child development. When postpartum depressive symptoms are additionally considered, these postnatal symptoms appeared to be more strongly associated with concurrent parent-child bonding, but also highlighted the individual relevance of prenatal depressive symptoms on child development. Parents reporting depressive symptoms during pregnancy and postpartum might struggle with adapting emotionally to the postpartum period. Professionals in perinatal care should take this perspective into account. Depressive symptoms, the experience of parenthood, and the developing relationship towards the child need to be addressed in the context of perinatal care. Preventive education on parental mental health and protective factors might help parents to identify depressive symptoms early on and encourage them to seek support by professionals or family and friends when needed. Further, our results underline the importance of including fathers’ perspective on and experience of parenthood in perinatal care contexts and research. This is highly relevant to identify potential risk factors for paternal adjustment and consequences for child development. As societal expectations and the lived realities of families shift towards models of parenting including increased childcare responsibilities for both parents, understanding mental health of individual family members in the context of the whole family system becomes increasingly relevant in both research and clinical practice.

## Electronic supplementary material

Below is the link to the electronic supplementary material.

**Supplementary Material 1**: Supplement Table 1 and Table 2

**Supplementary Material 2**: Supplement Table 3 and Table 4

**Supplementary Material 3**: Supplement Table 5

## Data Availability

The dataset analyzed during the current study is not publicly available due to legal and ethical constraints as the study’s informed consent did not include public sharing of participant data. The dataset is available from the corresponding author on reasonable request.

## Abbreviations

ASQ-3: Ages and Stages Questionnaire
C: Completers
DREAM: Dresden Study on Parenting, Work, and Mental Health (“DResdner Studie zu Elternschaft, Arbeit und Mentaler Gesundheit,” DREAM)
EPDS: Edinburgh Postnatal Depression Scale
F-SozU K-14: 14-item short form of the Perceived Social Support Questionnaire (Fragebogen zur sozialen Unterstützung)
NC: Non-completers
NP: No partner also participating
PBQ: Postpartum Bonding Questionniare
WP: With partner also participating
