# Supplementary material for: The mediating role of parent-child bonding for the prospective association of prenatal depressive symptoms with child development at 14 months postpartum
Source: BMC Pediatr. 2025 May 27;25:424. doi: 10.1186/s12887-025-05730-5 (PMC12107893; doi:10.1186/s12887-025-05730-5)
Supplement: Supplementary file 1 — Supplementary Material 1: Supplement Table 1 and Table 2 [file 12887_2025_5730_MOESM1_ESM.docx]

**Supplement Table 1**

*Comparison of parents with and without a partner also participating in this study using t-tests*

|  |  |  | *n*^a^ | *t^b^* | *df* | 95% BCa CI | | *p* |
| --- | --- | --- | --- | --- | --- | --- | --- | --- |
|  |  |  |  |  |  | lower | upper |  |
| Parental age | Mothers | NP | 317 | 3.58 | 529.46 | 0.40 | 1.50 | <.001 |
|  |  | WP | 849 |  |  |  |  |  |
|  | Fathers | NP | 27 | 2.10 | 27.96 | 0.22 | 3.92 | .050 |
|  |  | WP | 710 |  |  |  |  |  |
| Prenatal depressive symptoms (EPDS, T1) | Mothers | NP | 321 | 1.23 | 581.26 | -0.18 | 0.79 | .227 |
|  |  | WP | 857 |  |  |  |  |  |
|  | Fathers | NP | 27 | 0.96 | 27.43 | -0.72 | 2.48 | .373 |
|  |  | WP | 709 |  |  |  |  |  |
| Postpartum depressive symptoms (EPDS, T2) | Mothers | NP | 321 | 2.00 | 537.89 | 0.05 | 1.07 | .044 |
|  |  | WP | 857 |  |  |  |  |  |
|  | Fathers | NP | 27 | 1.07 | 27.36 | -0.53 | 2.40 | .286 |
|  |  | WP | 716 |  |  |  |  |  |
| Parent-child bonding (PBQ, T2) | Mothers | NP | 321 | 0.26 | 610.08 | -1.03 | 1.40 | .794 |
|  |  | WP | 854 |  |  |  |  |  |
|  | Fathers | NP | 27 | 1.95 | 27.22 | 0.12 | 7.74 | .060 |
|  |  | WP | 711 |  |  |  |  |  |
| Perceived social support (F-SozU K-14, T2) | Mothers | NP | 318 | -2.29 | 531.52 | -0.16 | -0.01 | .024 |
|  |  | WP | 853 |  |  |  |  |  |
|  | Fathers | NP | 26 | -0.40 | 28.24 | -0.22 | 0.15 | .672 |
|  |  | WP | 712 |  |  |  |  |  |

*Note*. EPDS: Edinburgh Postnatal Depression Scale; PBQ; Postpartum Bonding Questionnaire; F-SozU K-14: 14-item short form of the Perceived Social Support Questionnaire (Fragebogen zur sozialen Unterstützung); T1: during pregnancy; T2: 8 weeks postpartum; T3: 14 months postpartum NP: without a partner also participating; WP: with partner participating; ^a^Sample size may vary slightly due to missing information for individual variables; ^b^Welch correction for heterogeneity of variance.

**Supplement Table 2**

*Comparison of parents with and without a partner also participating in this study using Chi-square test of independence*

|  |  |  | *n*^a^ | *X^2^* | *df* | *p* |
| --- | --- | --- | --- | --- | --- | --- |
| Education | Mothers | NP | 321 | .07 | 1 | .795 |
|  |  | WP | 857 |  |  |  |
|  | Fathers | NP | 27 | 0.12 | 1 | .734 |
|  |  | WP | 709 |  |  |  |

*Note.* NP: without a partner also participating; WP: with partner participating.
