# Supplementary material for: The mediating role of parent-child bonding for the prospective association of prenatal depressive symptoms with child development at 14 months postpartum
Source: BMC Pediatr. 2025 May 27;25:424. doi: 10.1186/s12887-025-05730-5 (PMC12107893; doi:10.1186/s12887-025-05730-5)
Supplement: Supplementary file 2 — Supplementary Material 2: Supplement Table 3 and Table 4 [file 12887_2025_5730_MOESM2_ESM.docx]

**Supplement Table 3**

*Attrition analysis using t-tests*

|  |  |  | *n*^a^ | *t* | *df* | 95% CI | | *p* |
| --- | --- | --- | --- | --- | --- | --- | --- | --- |
|  |  |  |  |  |  | lower | upper |  |
| Parental age | Mothers^b^ | C | 1369 | 0.66 | 409 | -0.36 | 0.72 | .509 |
|  |  | NC | 301 |  |  |  |  |  |
|  | Fathers | C | 921 | 0.13 | 461 | -0.63 | 0.73 | .894 |
|  |  | NC | 280 |  |  |  |  |  |
| Prenatal depressive symptoms (EPDS, T1) | Mothers^b^ | C | 1380 | -2.97 | 401 | -1.43 | -0.29 | .003 |
|  |  | NC | 300 |  |  |  |  |  |
|  | Fathers | C | 923 | -1.97 | 1202 | -0.96 | 0.00 | .049 |
|  |  | NC | 281 |  |  |  |  |  |
| Postpartum depressive symptoms (EPDS, T2) | Mothers^b^ | C | 1314 | -2.45 | 205 | -1.48 | -0.16 | .015 |
|  |  | NC | 167 |  |  |  |  |  |
|  | Fathers | C | 861 | -1.59 | 1000 | -1.08 | 0.11 | .113 |
|  |  | NC | 141 |  |  |  |  |  |
| Parent-child bonding (PBQ, T2) | Mothers | C | 1293 | -0.11 | 1457 | -1.87 | 1.54 | .917 |
|  |  | NC | 166 |  |  |  |  |  |
|  | Fathers | C | 843 | -0.01 | 983 | -1.70 | 1.54 | .990 |
|  |  | NC | 142 |  |  |  |  |  |
| Perceived social support (F-SozU K-14, T2) | Mothers | C | 1375 | 1.19 | 1674 | -0.03 | 0.11 | .235 |
|  |  | NC | 301 |  |  |  |  |  |
|  | Fathers | C | 920 | 0.73 | 1196 | -0.05 | 0.12 | .464 |
|  |  | NC | 278 |  |  |  |  |  |

*Note*. EPDS: Edinburgh Postnatal Depression Scale; PBQ: Postpartum Bonding Questionnaire; F-SozU K-14: 14-item short form of the Perceived Social Support Questionnaire (Fragebogen zur sozialen Unterstützung). C: completer; NC: non-completer. T1: during pregnancy; T2: 8 weeks postpartum; T3: 14 months postpartum. ^a^Sample size may vary slightly due to missing information for individual variables; ^b^Welch correction for heterogeneity of variance.

**Supplement Table 4**

*Attrition analysis using Chi-square test of independence*

|  |  |  | *n*^a^ | *X^2^* | *df* | *p* |
| --- | --- | --- | --- | --- | --- | --- |
| Education | Mothers | C | 1382 | 20.31 | 1 | < .001 |
|  |  | NC | 301 |  |  |  |
|  | Fathers | C | 919 | 8.58 | 1 | .003 |
|  |  | NC | 284 |  |  |  |

*Note.* C: completer; NC: non-completer. ^a^Sample size may vary slightly due to missing information for individual variables.
