# Supplementary material for: The mediating role of parent-child bonding for the prospective association of prenatal depressive symptoms with child development at 14 months postpartum
Source: BMC Pediatr. 2025 May 27;25:424. doi: 10.1186/s12887-025-05730-5 (PMC12107893; doi:10.1186/s12887-025-05730-5)
Supplement: Supplementary file 3 — Supplementary Material 3: Supplement Table 5 [file 12887_2025_5730_MOESM3_ESM.docx]

**Supplement Table 5**

*Bivariate associations among the included variables between mothers and fathers.*

|  | Mothers | | | | | | | |
| --- | --- | --- | --- | --- | --- | --- | --- | --- |
| Fathers | 1 | 2 | 3 | 4 | 5 | 6 | 7 |  |
| 1. Prenatal depressive symptoms (EPDS, T1) | .127** | .089* | .044 | -.014 | .023 | -.068 | -.153*** |  |
| 2. Postpartum depressive symptoms (EPDS, T2) | .103** | .188*** | .157*** | -.087* | .001 | -.045 | -.164*** |  |
| 3. Parent-child bonding ^a^ (PBQ, T2) | .055 | .134*** | .224*** | -.137*** | -.065 | .071 | -.123** |  |
| 4. Child development (ASQ-3, T3) | .017 | -.040 | -.042 | .717*** | -.124** | .049 | .082* |  |
| 5. Parental age | .002 | .001 | -.040 | .005 | .640*** | -.009 | .000 |  |
| 6. Education | -.054 | -.069 | .052 | .039 | .083* | .263*** | .025 |  |
| 7. Perceived social support (F-SozU K-14, T2) | -.141*** | -.078 | .001 | .069 | -.053 | .001 | .335*** |  |
| *Note*. Based on data from 1,178 couples. EPDS: Edinburgh Postnatal Depression Scale; PBQ: *Postpartum*Bonding Questionnaire**;** ASQ-3: Ages and Stages Questionnaire-3; F-SozU K-14: 14-item short form of the Perceived Social Support Questionnaire (Fragebogen zur sozialen Unterstützung); T1: during pregnancy; T2: 8 weeks postpartum; T3: 14 months postpartum. ^a^A higher score in parent-child bonding indicates more bonding impairment. * *p* < .05; ** *p* < .01; *** *p* < .001. | | | | | | | | |
